# Disturbance of the OPG/RANK/RANKL pathway and systemic inflammation in COPD patients with emphysema and osteoporosis

**DOI:** 10.1186/1465-9921-12-157

**Published:** 2011-12-16

**Authors:** Peng Bai, Yongchang Sun, Jianmin Jin, Jia Hou, Ran Li, Qing Zhang, Yang Wang

**Affiliations:** 1 Department of Respiratory Medicine, Beijing Tongren Hospital, Capital Medical University, Beijing, China; 2 Department of Radiology, Beijing Tongren Hospital, Capital Medical University, Beijing, China; 3 Central Laboratory, Beijing Tongren Hospital, Capital Medical University, Beijing, China

**Keywords:** chronic obstructive pulmonary disease, pulmonary emphysema, osteoporosis, cytokine, OPG/RANK/RANKL

## Abstract

**Background:**

Osteoporosis is one of the systemic features of COPD. A correlation between the emphysema phenotype of COPD and reduced bone mineral density (BMD) is suggested by some studies, however, the mechanisms underlying this relationship are unclear. Experimental studies indicate that IL-1β, IL-6 and TNF-α may play important roles in the etiology of both osteoporosis and emphysema. The OPG/RANK/RANKL system is an important regulator of bone metabolism, and participates in the development of post-menopausal osteoporosis. Whether the OPG/RANK/RANKL pathway is involved in the pathogenesis of osteoporosis in COPD has not been studied.

**Methods:**

Eighty male patients (current or former smokers) completed a chest CT scan, pulmonary function test, dual x-ray absorptiometry measurements and questionnaires. Among these subjects, thirty patients with normal BMD and thirty patients with low BMD were selected randomly for measurement of IL-1β, IL-6, TNF-α (flow cytometry) and OPG/RANK/RANKL (ELISA). Twenty age-matched healthy volunteers were recruited as controls.

**Results:**

Among these eighty patients, thirty-six had normal BMD and forty-four had low BMD. Age, BMI and CAT score showed significant differences between these two COPD groups (*p *< 0.05). The low-attenuation area (LAA%) in the lungs of COPD patients was negatively correlated with lumbar vertebral BMD (r = 0.741; *p *< 0.0001). Forward logistic regression analysis showed that only LAA% (*p *= 0.005) and BMI (*p *= 0.009) were selected as explanatory variables. The level of IL-1β was significantly higher in the COPD patients as compared to the normal controls (*p *< 0.05), but the difference between the two COPD groups did not reach significance. The levels of IL-6 and TNF-α among the three groups were significantly different (*p *< 0.05). The level of RANKL and the RANKL/OPG ratio were significantly higher in COPD patients with low BMD compared to those with normal BMD and the normal controls (*p *< 0.05), and correlated negatively with lumbar vertebral BMD, but positively with LAA%.

**Conclusions:**

Radiographic emphysema is correlated with low BMD in current and former smokers with COPD. IL-1β, IL-6, TNF-α, and the osteoporosis-related protein system OPG/RANK/RANKL may have some synergetic effects on emphysema and bone loss in COPD.

## Background

Chronic obstructive pulmonary disease (COPD) is recognized as a highly prevalent condition which causes significant morbidity and mortality [1], and commonly associated with many extra-pulmonary abnormalities such as cardiovascular disease, cachexia, skeletal muscle wasting, and anemia [2,3]. Osteoporosis is one of the most important systemic comorbidities in COPD, which increases the risk of osteoporotic fractures, and carries a heavy economic burden [4]. It has been reported that bone mineral density (BMD) is lower in COPD patients than in healthy subjects [5-7]. Studies have shown that low BMD in COPD patients is related to some clinical and physiological indices, such as lung function (FEV_1_), low body weight and decreased fat-free mass [8,9]. BMD can be measured by Dual X-ray Absorptiometry (DXA). With the advantages of high precision, short scan times, and low radiation dose, DXA provides a noninvasive method of diagnosing osteoporosis and guiding decisions about treatment [10,11].

Emphysema is a primary imaging manifestation in COPD patients, and has been recognized as an important phenotype of COPD. The low-attenuation area (LAA) in the lungs evaluated by chest CT images has been widely used to quantitatively assess pulmonary emphysema [12]. However, the association between emphysema and osteoporosis in COPD patients and its possible underlying mechanism are still unclear. In fact, similarities between parenchymal emphysema and osteoporosis, including the loss of extracellular matrix and common association with inflammatory mediators, alludes to a potential mechanistic link between the two processes.

Patients with COPD have evidence of systemic inflammation, which may be responsible for some comorbidities. Levels of inflammatory cytokines such as IL-1β, IL-6 and TNF-α are increased in the systemic circulation of COPD patients [13-15]. Inflammatory cytokines, including IL-1β, IL-6 and TNF-α, are also responsible for the characteristic loss of bone density in osteoporosis through their effect on osteoclast activity [16,17]. The osteoporosis related-protein triad osteoprotegerin (OPG)/receptor activator of NF-κB (RANK)/RANK ligand (RANKL) has been identified as an important regulator of bone metabolism and remodeling. This system has been shown to interact with IL-1β, IL-6 and TNF-α, and play important roles in the occurrence of postmenopausal osteoporosis [18,19]. This raises the intriguing possibility that the OPG/RANK/RANKL pathway may also be involved in the development of osteoporosis in COPD.

Our hypothesis was that the extent of emphysema was associated with the severity of osteoporosis in COPD patients, and that pro-inflammatory cytokines and the OPG/RANK/RANKL system were altered and correlated with osteoporosis in these patients.

## Methods

### Subjects

Eighty male COPD patients diagnosed as clinically stable were enrolled from January 2010 to May 2011. Female patients were excluded from the study to avoid the influence of postmenopausal osteoporosis. COPD was diagnosed according to the criteria of the Global Initiative for Chronic Obstructive Lung Disease (GOLD) [2]. All patients were more than 40 years old, and were current or former smokers. The exclusion criteria were as follows: (1) acute exacerbation of COPD in the last 3 months, (2) history of respiratory diseases other than COPD, (3) history of chest surgery, (4) occurrence of malignancy within the previous five years, (5) history of bone disease, (6) current and/or previous inhale, oral or intravenous corticosteroid therapy, (7) other systemic diseases, such as renal insufficiency and thyroid diseases, or medication which may influence bone metabolism within the previous one year.

The study was approved by the local ethics committee of Beijing Tongren Hospital, Capital Medical University (TRECKT 2008-14). All subjects provided informed consent to join the study.

On the recruiting day, each subject completed a chest CT scan, prebronchodilator and post-bronchodilator spirometry and plethysmography, and DXA measurements of BMD of the femoral neck and lumbar spine. Recording of demographic parameters and medical history, evaluation of quality of life, and collection of blood samples were also completed on that day.

### Analysis of LAA

CT scans were performed using a multidetector row scanner (Brilliance 64, Phillips, Eindhoven, Netherlands) with technical parameters of 120 kVp, slice thicknesses of 1.0 mm, and a thread interval of 11.08 mm. During scanning, subjects were asked to hold their breath after a deep inspiration in the supine position.

The percentage of LAA (LAA%) was measured automatically using specified software in the Extended Brilliance Workspace to quantitatively evaluate pulmonary emphysema. All CT images from the upper margin of the aortic arch to the diaphragm were used to calculate the LAA%. Lung fields were defined as areas with a CT scan density of less than -200 Hounsfield units (HU), and the threshold between the LAA and normal lung density was defined as -950 HU. The LAA% was calculated as (number of LAA pixels in all slices)/(total lung area)×100 (%). The measurements of LAA% were performed independently by an experienced radiologist, who was blinded to the subjects' clinical information.

### Measurements of BMD

BMD at the lumbar spine (L1-L4) and the bilateral femoral neck was measured by Dual X-Ray Absorptiometry (Lunar prodigy, GE Healthcare, London, United Kingdom). BMD is reported as an absolute value (g/cm^2^) and a T score, which represents the number of standard deviations from a young, sex- and ethnic group-specific reference mean. According to the definition of the World Health Organization (WHO), T scores are used as the basis for diagnosis as follows: normal bone mineral density: T score greater than -1 at both sites (femoral neck and lumbar spine), osteopenia: T score less than or equal to -1 but greater than -2.5 at either site, osteoporosis: T score less than or equal to -2.5 at either site [20]. On the basis of this standard, we classified the subjects into two groups: COPD with normal BMD (T score greater than -1 at both sites) (36 patients) and COPD with low BMD (osteopenia/osteoporosis, T score less than or equal to -1 at either site) (44 patients).

### Measurements of IL-1β, IL-6, TNF-α and OPG/RANK/RANKL

Among the eighty subjects, thirty patients with normal BMD and thirty patients with low BMD were randomly selected for the measurement of serum cytokines. Twenty healthy volunteers (current or former smokers, mean age: 58.4 ± 7.1), whose BMD at the lumbar spine (L1-L4) and the bilateral femoral neck measured by DXA were normal, were recruited as normal controls. The smoking index showed no significant difference among these three groups (*p *> 0.05). The exclusion criteria were the same as those used for the COPD subjects. Fasting blood samples (approximately 20 mL) were collected by venipuncture in plain tubes. Sera were obtained by centrifugation at 1000 × g for 5 min at room temperature. The samples were stored at -80°C until analysis. The levels of IL-1β, IL-6, and TNF-α in serum samples were evaluated by flow cytometry (CBA Kit, BD Bioscience, USA). OPG/RANK/RANKL concentrations were measured by ELISA (Catalog No.CSB-E04692h, 14931h, 08157h; Cusabio Biotech Co., Ltd, USA). Assays were performed according to the manufacturer's protocol.

### Evaluation of Clinical Characteristics

Dyspnea was rated using the American Thoracic Society MMRC dyspnea score [21].

Six-Minute Walking Distance (6MWD): The patients walked in the same hospital hallway without oxygen supplementation encouraged by a technician. Processing was consistent with the ATS guidelines for the six minute walk test [22].

BODE indices were calculated as the sum score proposed by Celli et al. [23], based on the following four variables: (1) FEV_1 _percentage of predicted (≥ 65%, score 0; 50% to 64%, score 1; 36% to 49%, score 2; and ≤ 35%, score 3); (2) Six-minute walking distance (6MWD) (≥ 350 m, score 0; 250 to 349 m, score 1; 150 to 249 m, score 2; ≤149 m, score 3); (3) MMRC dyspnea score (0 to 1, score 0; 2, score 1; 3, score 2; and 4, score 3); and (4) body mass index (BMI) (> 21 kg/m^2^, score 0; ≤ 21 kg/m^2^, score 1). Possible total scores ranged from 0 to 10.

All patients were assessed by interview using the COPD Assessment Test (CAT) [24], and the scores were recorded.

### Data analysis

All statistical analyses were performed using a statistical software package (Statistics Package for the Social Sciences, SPSS 17.0, Inc., Chicago, IL, USA). Data are expressed as the mean ± SD. Comparisons of data between two groups (COPD with normal BMD and COPD with low BMD) were performed by T-test (for normal distribution parameters) and Mann-Whitney U test (for abnormal distribution parameters). Since the subjects' age among control, COPD with normal BMD and COPD with low BMD groups had significant difference (*p *< 0.05), the comparisons of serum cytokines were performed by analysis of covariance. The relationships among continuous parameters were evaluated by the multiple linear regression analyses. Based on the grouping of BMD, forward logistic regression analyses were performed to evaluate the contribution of LAA% and other parameters which could cause a significant difference between these two groups. A p value of < 0.05 was considered significant.

## Results

### Demographic parameters, pulmonary function results and quality of life score of eighty COPD patients

Comparisons of age, BMI, FEV_1_/FVC, FEV_1 _% predicted, CAT score and BODE index between the two COPD groups (COPD with normal BMD and COPD with low BMD) are shown in Table 1. Age, BMI and CAT score showed significant differences between these two groups, but there was no significant difference in FEV_1_/FVC, FEV_1 _% predicted, smoking index and BODE index.

**Table 1:** Comparison of demographic parameters, pulmonary function results and Quality of Life Score between two COPD groups

Variables	COPD with normal BMD (n = 36)	COPD with low BMD (n = 44)	p-value
Age(yr)	65.5 ± 5.1	67.8 ± 5.2	0.049
BMI(kg/m^2^)	26.1 ± 3.6	23.5 ± 2.9	< 0.001
Smoking index(pack-yr)	28.1 ± 26.0	26.7 ± 21.6	0.79
CAT score	15.8 ± 9.6	21.0 ± 8.5	0.01
BODE index	3.3 ± 1.1	4.1 ± 1.2	0.26
FEV_1_/FVC(%)	57.0 ± 10.1	53.9 ± 9.6	0.17
FEV_1 _% predicted(%)	58.4 ± 19.2	52.8 ± 17.3	0.18

Data are mean (SD) unless otherwise stated.

COPD: chronic obstructive pulmonary disease, BMD: bone mineral density, BMI: body mass index, CAT: COPD Assessment Test, BODE index: body mass index, airflow obstruction, dyspnea, and exercise capacity index, FEV1: forced expiratory volume in one second

### CT scan measurements for pulmonary emphysema

As a quantitative assessment of pulmonary emphysema, the mean value of LAA% for all 80 COPD patients was 24.66 ± 12.90%. Values of LAA% were 18.67 ± 10.00% and 29.57 ± 13.03% for COPD with normal and low BMD groups, respectively, with a significant difference between groups (*p *< 0.0001).

### BMD in the femoral neck and lumbar spine

**Figure 1:**
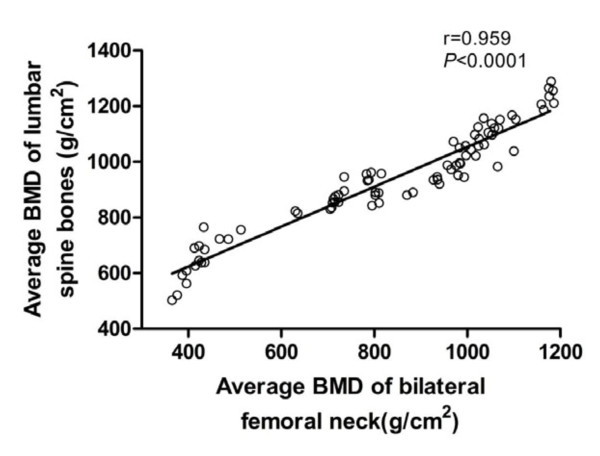
**Relationship between the average BMD of lumbar spine bones and bilateral femoral necks**. The average BMD of femoral neck showed a good correlation with lumbar vertebra (Average BMD of lumbar spine bones = 0.718 × Average BMD of bilateral femoral necks +336.46;r = 0.959; p < 0.0001).

To reduce the influence of BMD variability among vertebral levels and between bilateral femoral necks, for each patient, the mean BMD of four lumbar vertebrae and of both femoral necks were used as the final results. The mean BMD of the femoral neck showed a good correlation with that of the lumbar vertebrae (r = 0.959; *p *< 0.0001), a measurement which has been widely used for the assessment of BMD (Figure 1). Therefore, we used lumbar vertebral BMD to further analyze the correlation of BMD with anthropometric parameters, quality of life score, and LAA%.

### Serum level of pro-inflammatory cytokines and OPG/RANK/RANKL

Serum levels of IL-1β, IL-6, TNF-α and OPG/RANK/RANKL were compared among the three groups (normal control, COPD with normal BMD and COPD with low BMD) (Table 2). For adjustment of the effect of age, analysis of covariance was used (age as the covariance).

**Table 2:** Comparison of IL-1β,IL-6, TNF-αand OPG/RANK/RANKL levels among three groups

Variables	normal control (n = 20)	COPD with normal BMD (n = 30)	COPD with low BMD (n = 30)
IL-1β (fg/ml)	122.21 ± 35.14	186.83 ± 104.87†	192.94 ± 115.82†
IL-6(fg/ml)	354.37 ± 83.14	421.52 ± 125.78†	483.73 ± 110.38†‡
TNF-α (fg/ml)	193.09 ± 81.20	261.28 ± 96.09†	324.37 ± 141.29†‡
OPG(pg/ml)	236.50 ± 38.76	240.43 ± 65.77	257.97 ± 86.80
RANK(pg/ml)	82.28 ± 29.76	73.90 ± 50.41	2.24 ± 0.80 †‡

Data are mean (SD) unless otherwise stated.

†p < 0.05 compared with the normal control group.

‡p < 0.05 compared with the COPD with normal BMD group

COPD: chronic obstructive pulmonary disease, BMD: bone mineral density, IL: interleukin, TNF-α:tumor necrosis factor α, OPG: osteoprotegerin, RANK: receptor activator of NF-κB, RANKL: RANK ligand

**Figure 2:**
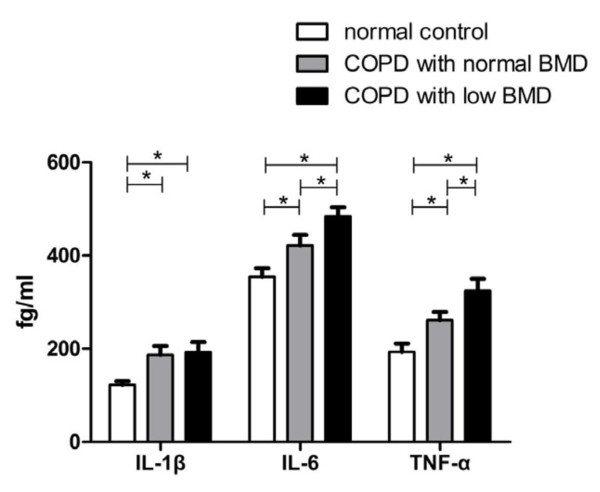
**Comparison of serum IL-1β, IL-6, TNF-α levels among three groups**. *p < 0.05. Abbreviations: IL: interleukin, TNF-α: tumor necrosis factor α.

**Figure 3:**
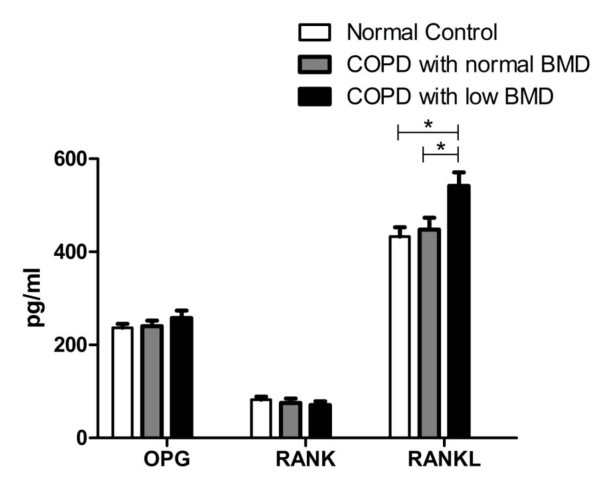
**Comparison of serum OPG/RANK/RANKL levels among three groups**. *p < 0.05. Abbreviations: OPG: osteoprotegerin, RANK: receptor activator of NF-κB, RANKL: RANK ligand.

**Figure 4:**
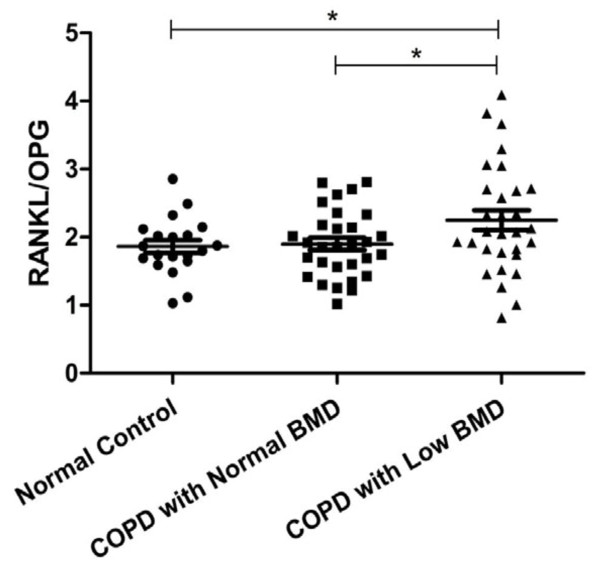
**Comparison of RANKL/OPG among three groups**. *p < 0.05. Abbreviations: OPG: osteoprotegerin, RANKL: receptor activator of NF-κB ligand.

The level of IL-1β was significantly higher in the COPD patients as compared to the normal controls (*p *< 0.05), but the difference between the two COPD groups did not reach significance. The levels of IL-6 and TNF-α among the three groups were significantly different (*p *< 0.05) (Figure 2). No significant difference existed in the level of OPG and RANK among the three groups, but the level of RANKL (Figure 3) and the ratio of RANKL/OPG (R/O) (Figure 4) were remarkably higher in COPD patients with low BMD compared to those with normal BMD and the normal controls (*p *< 0.05).

### Correlation of BMD with pro-inflammatory cytokines and OPG/RANK/RANKL concentration

Multiple linear regression analyses showed that, with the adjustment of age, among IL-1β, IL-6, TNF-α, OPG, RANK, RANKL and the ratio of RANKL/OPG, only the level of RANKL (r = -0.256, *p *= 0.015) and the ratio of RANKL/OPG (r = -0.258, *p *= 0.015) had a significant negative correlation with lumbar vertebral BMD.

### Correlation of pulmonary emphysema severity (LAA%) with proinflammatory cytokines and OPG/RANK/RANKL concentration

Multiple linear regression analyses showed that, with the adjustment of age, among IL-1β, IL-6, TNF-α, OPG, RANK, RANKL and the ratio of RANKL/OPG, only the level of RANKL (r = 0.216, *p *= 0.043) and the ratio of RANKL/OPG (r = 0.213, *p *= 0.041) had a significant positive correlation with LAA%.

### Correlation of BMD with pulmonary emphysema severity (LAA%) and other variables

Pearson product-moment correlation analyses revealed that LAA% had a significant negative correlation with lumbar vertebral BMD (r = -0.741; *p *< 0.0001). Because the complex relationship between COPD and osteoporosis is confounded by the overlap of common risk factors, a forward logistic regression analysis was performed, based on the grouping of BMD and factors which showed a significant difference between the two COPD groups (i.e., LAA%, age, BMI and CAT) as independent variables. This showed that only LAA% (*p *= 0.005) and BMI (*p *= 0.009) were explanatory variables, indicating that LAA% is the strongest predictor of osteopenia/osteoporosis.

## Discussion

COPD has been recognized as a systemic disease which also involves several extra-pulmonary features. Osteoporosis is one of the major systemic comorbidities of COPD, which is more prevalent among COPD patients than healthy subjects [5,8]. The etiology of COPD-related osteoporosis is almost certainly multifactorial. With the exception of BMI [6-9], the effects of other factors which have been reported to be responsible for the reduced bone density in patients with COPD, such as age [6], poor quality of life [6], FEV_1 _[6,9], smoking [6], and corticosteroid therapy [6,8,25] remain controversial. In our study, BMI was lower in the COPD with low BMD group than the COPD with normal BMD group, and logistic regression analysis showed that BMI was an independent predictor for BMD. This result is in accordance with previous studies [6-9]. In our study, patients in both of the two COPD groups were older than the control, and the COPD patients with low BMD were older than those with normal BMD, the difference among these three groups being significant (*p *< 0.05). But age was not an independent variable in logistic regression analysis. The effect of age on COPD-related bone loss is still indefinite, and age may influence the serum level of pro-inflammatory cytokines. Therefore, when performing the data analysis, we used analysis of covariance and multiple linear regression to correct the influence of age. BMI and age are both correlated with quality of life. CAT score and BODE index are two new indices used to evaluate the quality of life for COPD patients. The trend in our study showed that patients with lower BMD had higher CAT scores (a relationship which was statistically significant) and BODE index (which did not reach statistical significance), indicating a deterioration in the quality of life. This result agrees with the findings of Iqbal et al. [6], showing that BMD correlated weakly with activity evaluated by St George's respiratory questionnaire (SGRQ), whereas Katsura and Kida [7] and Kjensli et al. [8] did not find a significant correlation with activity evaluated by the 6MWD. Several studies have found no significant relationships between bone density and airflow obstruction [7,26], and these are in agreement with our study: FEV_1_/FVC and FEV_1 _% predicted showed no significant difference between the two COPD groups. In the study of Duckers et al., over 80% of the male COPD with osteopenia/osteoporosis had mild to moderate severity airways obstruction(GOLD I and II) [27]. Further studies are needed to investigate the relationships between bone density and airflow obstruction.

In the present study, we demonstrated that LAA%, which reflects the extent of emphysema, correlated significantly with reduced bone density. In addition, logistic regression analysis showed that only LAA% and BMI could predict bone density in male COPD patients. It means that, in addition to the effect of BMI, LAA% is also an independent predictor of bone loss and responsible for the difference which we has found between COPD patients with low BMD and those with normal BMD. This result is in accordance with that of Ohara and coworkers, who found a linear correlation between emphysema and absolute BMD of the thoracic and L1 vertebrae measured by quantitative CT [28]. Recently, a study by Bon and coworkers also showed that radiographic emphysema was a strong, independent predictor of low BMD in current and former smokers with COPD [20]. These results provide support for the hypothesis that emphysema itself could be a risk factor for bone loss.

Destruction of the lung parenchyma may be associated in some way with the mechanism of the destruction of bone structure. The underlying mechanisms are speculative and may be related to systemic inflammatory factors acting synchronously on the lung parenchyma and bone matrix. IL-1β, IL-6 and TNF-α are all well-established inflammatory cytokines involved in the systemic inflammation of COPD. In our study, the serum levels of IL-1β, IL-6 and TNF-α in COPD patients were all significantly higher than in controls, supporting the existence of systemic inflammation in COPD. Bon and coworkers have demonstrated an association between distinct serum inflammatory mediators and quantitative CT measurements of emphysema [29]. Simultaneously, IL-1β, IL-6 and TNF-α have been shown to be important modulators of bone metabolism and remodeling, by stimulating osteoclast differentiation in a synergistic fashion [16]. These cytokines may not only regulate osteoclastogenesis via their effects on stromal cells, but also act directly on osteoclasts and their precursors [30]. In addition, they also interact with the osteoporosis-related OPG/RANK/RANKL protein system [18].

Recently, the OPG/RANK/RANKL system has been shown to have pleiotropic effects on both bone metabolism [31] and the immune system [32], and has led to a new molecular perspective on osteoclast biology and bone homeostasis. RANKL and OPG, synthesized by stromal cells/osteoblasts, have been identified as two principal cytokines controlling osteoclastic differentiation and activation. RANK, localized at the cell surface of mature osteoclasts and osteoclastic precursors, is the third protagonist [33,34]. The predominant role of RANKL in bone physiology is the stimulation of osteoclastic differentiation/activation and the inhibition of osteoclast apoptosis [35]. Binding of RANKL to RANK stimulates (1) differentiation of osteoclastic precursors into mature osteoclasts, and (2) activation of mature osteoclasts. OPG acts as a decoy receptor for RANKL and thus down-regulates RANKL signaling through RANK. It represents an endogenous decoy receptor that neutralizes the biological effects of all forms of RANKL and thus acts as an inhibitor of bone resorption [33,34]. In fact, bone remodeling appears to be mainly controlled by the balance of RANKL/OPG. Any modification in the RANKL-to-OPG ratio can induce either excessive bone resorption or, in contrast, excessive bone formation [35]. Dysregulation of their relative expression can lead to pathological conditions such as osteopenia/osteoporosis, bone tumor-associated osteolysis, immune disease, or cardiovascular pathology [16]. It has been demonstrated both in vivo and in vitro, that IL-1β, IL-6 and TNF-α, which are the products of stromal cells and monocytes, increase production of both RANKL and OPG. The dominant outcome of these three cytokines is a net increase in RANKL activity, which leads to bone loss. In addition, increases of RANKL and OPG can also up-regulate the expression of IL-6 and TNF-α [19], which may enhance the systemic inflammation of COPD.

In the present study, we found that the serum levels of IL-6, TNF-α, RANKL and the ratio of RANKL/OPG were significantly higher in the COPD with low BMD group than in the other two groups (controls and normal BMD); the level of IL-1β was also higher in the low BMD group than in the normal BMD group, but did not reach statistical significance. Only the level of RANKL and the ratio of RANKL/OPG were significantly correlated with lumbar vertebral BMD (negative) and LAA% (positive).

It is well known that smoking can not only influence the serum level of inflammatory cytokines (included IL-1β, IL-6, TNF-α and OPG/RANK/RANKL system), but also accelerate the bone loss. In our study, patients and the control were all current or former smokers, and the smoking index showed no significant difference among these three groups (*p *> 0.05). But further studies involving both smoking and non-smoking subjects are needed.

In the study of Duckers et al., the serum level of OPG was greater in COPD patients who combined with osteopenia/osteoporosis than those without low BMD and related inversely to hip BMD, but the level of RANKL and the ratio of RANKL/OPG were not determined [27]. In our study, the level of OPG did not significantly differ among these three groups. However, the study of Eagan and coworkers found that the level of OPG was significantly lower in COPD patients [36]. Both our study and that of Eagan et al. showed that, in COPD patients, the balance of the OPG/RANK/RANKL system is destroyed and manifests a dominant trend for RANKL. Thus, we assume that systemic inflammation may increase the serum levels of inflammatory cytokines (especially, IL-6 and TNF-α), which then disturb the balance of the OPG/RANK/RANKL system and result in a dominant trend for RANKL. This trend may cause the bone loss and osteopenia/osteoporosis seen among COPD patients. Further studies will be necessary to validate our hypothesis.

There are several limitations to our study. Firstly, the subjects were all male. Female patients, who have been reported to have a much higher incidence of osteopenia/osteoporosis, were not involved. Second, this study did not include patients who never smoked. Third, the size of the study population was relatively small. Further studies, involving more patients, including both males and females, as well as smoking and non-smoking subjects, are necessary.

## Conclusions

In conclusion, our study demonstrated that the extent of pulmonary emphysema was significantly correlated with reduced bone density, and for the first time to our knowledge, that the level of RANKL and the RANKL/OPG ratio were significantly higher in COPD patients with low BMD compared to those with normal BMD, and correlated negatively with lumbar vertebral BMD, but positively with LAA%. Although osteoporosis results from multiple causes such as age, smoking, malnutrition, and steroid treatment, the systemic inflammation of COPD may contribute directly to osteoporosis to some extent. There may be congenerous molecular mechanisms involved in the destruction of both lung parenchyma and bone structure, and inflammatory cytokines, such as IL-1β, IL-6 and TNF-α, as well as the osteoporosis related protein triad OPG/RANK/RANKL, may have some synergetic effects on both emphysema and bone loss. Further studies are needed to explore the mechanisms underlying COPD-related bone loss.

## Abbreviations

COPD: chronic obstructive pulmonary disease; BMD: bone mineral density; DXA: Dual X-ray Absorptiometry; LAA: low-attenuation area; OPG: osteoprotegerin; RANK: receptor activator of NF-κB; RANKL: RANK ligand; FEV_1_: forced expiratory volume in one second; FVC: forced vital capacity; BMI: body mass index; 6MWD: six-minute walking distance; CAT: COPD Assessment Test; GOLD: the Global Initiative for Chronic Obstructive Lung Disease.

## Competing interests

The authors declare that they have no competing interests.

## Authors' contributions

PB completed the recruiting of patients, performed the collection and analysis of all data and blood samples, and was a major contributor in writing the manuscript.

YS was the primary investigator of this study and was a major contributor in writing the manuscript.

JJ and JH were two major contributors in writing the manuscript.

RL completed the recruiting of patients, performed the collection of clinical data.

QZ performed the evaluation of low-attenuation area (LAA) in the lungs on chest CT images.

YW performed the laboratory-based assays.

All authors read and approved the final manuscript.

## References

[B1] Lopez AD Shibuya K Rao C Mathers CD Hansell AL Held LS Schmid V Buist S Chronic obstructive pulmonary disease: current burden and future projections Eur Respir J 2006 27 397 412 10.1183/09031936.06.00025805 16452599

[B2] Rabe KF Hurd S Anzueto A Barnes PJ Buist SA Calverley P Fukuchi Y Jenkins C Rodriguez-Roisin R Van Weel C Zielinski J Global strategy for the diagnosis, management, and prevention of chronic obstructive pulmonary disease: Gold executive summary Am J Respir Crit Care Med 2007 176 532 555 10.1164/rccm.200703-456SO 17507545

[B3] Agusti AG Soriano JB COPD as a systemic disease COPD 2008 5 133 138 10.1080/15412550801941349 18415812

[B4] National Osteoporosis Foundation. Physician's guide to prevention and treatment of osteoporosis 2003 Washington, DC. National Osteoporosis Foundation

[B5] Agusti AG Noguera A Sauleda J Sala E Pons J Busquets X Systemic effects of chronic obstructive pulmonary disease Eur Respir J 2003 21 347 360 10.1183/09031936.03.00405703 12608452

[B6] Iqbal F Michaelson J Thaler L Rubin J Roman J Mark S Declining bone mass in men with chronic pulmonary disease: contribution of glucocorticoid treatment, body mass index, and gonadal function Chest 1999 116 1616 1624 10.1378/chest.116.6.1616 10593785

[B7] Katsura H Kida K A comparison of bone mineral density in elderly female patients with COPD and bronchial asthma Chest 2002 122 1949 1955 10.1378/chest.122.6.1949 12475832

[B8] Kjensli A Mowinckel P Ryg MS Falch JA Low bone mineral density is related to severity of chronic obstructive pulmonary disease Bone 2007 40 493 497 10.1016/j.bone.2006.09.005 17049326

[B9] Vrieze A de Greef MH Wy'kstra PJ Wempe JB Low bone mineral density in COPD patients related to worse lung function, low weight and decreased fat-free mass Osteoporos Int 2007 18 1197 1202 10.1007/s00198-007-0355-7 17347789

[B10] Mineo TC Ambrogi V Mineo D Fabbri A Fabbrini E Massoud R Bone mineral density improvement after lung volume reduction surgery for severe emphysema Chest 2005 127 1960 1966 10.1378/chest.127.6.1960 15947308

[B11] Blake GM Knapp KM Fogelman I Dual X-ray absorptiometry-clinical evaluation of a new cone-beam system Calcif Tissue Int 2005 76 113 120 10.1007/s00223-004-0080-6 15645160

[B12] Madani A Zanen J de Maertelaer V Gevenois PA Pulmonary emphysema: objective quantification at multi-detector row CT; comparison with macroscopic and microscopic morphometry Radiology 2006 238 1036 1043 10.1148/radiol.2382042196 16424242

[B13] Garcia-Rio F Miravitlles M Soriano JB Muñoz L Duran-Tauleria E Sánchez G Sobradillo V Ancochea J EPI-SCAN Steering Committee Systemic inflammation in chronic obstructive pulmonary disease: a population-based study Respiratory Research 2010 11 63 20500811 10.1186/1465-9921-11-63 PMC2891677

[B14] Barnes PJ Celli BR Systemic manifestation and comorbidities of COPD Eur Respir J 2009 33 1165 1185 10.1183/09031936.00128008 19407051

[B15] Hurst JR Perera WR Wilkinson TM Donaldson GC Wedzicha JA Systemic and upper and lower airway inflammation at exacerbation of chronic obstructive pulmonary disease Am J Respir Crit Care Med 2006 173 71 78 10.1164/rccm.200505-704OC 16179639

[B16] Gregory R Mundy MD Osteoporosis and Inflammation Nutrition Reviews 2007 65 S147 S151 10.1301/nr.2007.dec.S147-S151 18240539

[B17] McLean RR Proinflammatory cytokines and osteoporosis Curr Osteoporos Rep 2009 7 134 139 10.1007/s11914-009-0023-2 19968917

[B18] Kwan TS Padrines M Théoleyre S Heymann D Fortun Y IL-6, RANKL, TNF-alpha/IL-1: interrelations in bone resorption pathophysiology Cytokine Growth Factor Rev 2004 15 49 60 10.1016/j.cytogfr.2003.10.005 14746813

[B19] Théoleyre S Wittrant Y Tat SK Fortun Y Redini F Heymann D The molecular triad OPG/RANK/RANKL: involvement in the orchestration of pathophysiological bone remodeling Cytokine Growth Factor Rev 2004 15 457 475 10.1016/j.cytogfr.2004.06.004 15561602

[B20] Bon J Fuhrman CR Weissfeld JL Duncan SR Branch RA Chang CH Zhang Y Leader JK Gur D Greenspan SL Sciurba FC Radiographic Emphysema Predicts Low Bone Mineral Density in a Tobacco-exposed Cohort Am J Respir Crit Care Med 2011 183 885 890 10.1164/rccm.201004-0666OC 20935108 PMC3086755

[B21] American Thoracic Society Surveillance for respiratory hazards in the occupational setting Am Rev Respir Dis 1982 126 952 956 7149469

[B22] ATS Committee on Proficiency Standards for Clinical Pulmonary Function Laboratories ATS statement: guidelines for the six minute walk test Am J Respir Crit Care Med 2002 116 111 117 10.1164/ajrccm.166.1.at1102 12091180

[B23] Celli BR Cote CG Marin JM Casanova C Montes de Oca M Mendez RA Plata P The body-mass index, airflow obstruction, dyspnea, and exercise capacity index in chronic obstructive pulmonary disease N Engl J Med 2004 350 1005 1012 10.1056/NEJMoa021322 14999112

[B24] Jones PW Harding G Berry P Wiklund I Chen WH Leidy NK Development and first validation of the COPD Assessment Test Eur Respir J 2009 34 648 654 10.1183/09031936.00102509 19720809

[B25] Lee TA Weiss KB Fracture risk associated with inhaled corticosteroid use in chronic obstructive pulmonary disease Am J Respir Crit Care Med 2004 169 855 859 10.1164/rccm.200307-926OC 14711795

[B26] Bolton CE Ionescu AA Shiels KM Pettit RJ Edwards PH Stone MD Nixon LS Evans WD Griffiths TL Shale DJ Associated loss of fat-free mass and bone mineral density in chronic obstructive pulmonary disease Am J Respir Crit Care Med 2004 170 1286 1293 10.1164/rccm.200406-754OC 15374843

[B27] Duckers JM Evans BA Fraser WD Stone MD Bolton CE Shale DJ Low bone mineral density in men with chronic obstructive pulmonary disease Respiratory Research 2011 12 101 10.1186/1465-9921-12-101 21812978 PMC3161864

[B28] Ohara T Hirai T Muro S Haruna A Terada K Kinose D Marumo S Ogawa E Hoshino Y Niimi A Chin K Mishima M Relationship between pulmonary emphysema and osteoporosis assessed by CT in patients with COPD Chest 2008 134 1244 1249 10.1378/chest.07-3054 18641115

[B29] Bon JM Leader JK Weissfeld JL Coxson HO Zheng B Branch RA Kondragunta V Lee JS Zhang Y Choi AMK Lokshin AE Kaminski N Gur D Sciurba FC The influence of radiographic phenotype and smoking status on peripheral blood biomarker patterns in chronic obstructive pulmonary disease PLoS ONE 4 8 e6865 10.1371/journal.pone.0006865 PMC2730536 19718453

[B30] Ragab AA Nalepka JL Bi Y Greenfield EM Cytokines synergistically induce osteoclast differentiation: support by immortalized or normal calvarial cells Am J Physiol Cell Physiol 2002 283 C679 687 12176725 10.1152/ajpcell.00421.2001

[B31] Horowitz MC Xi Y Wilson K Kacena MA Control of osteoclastogenesis and bone resorption by members of the TNF family of receptors and ligands Cytokine Growth Factor Rev 2001 12 9 18 10.1016/S1359-6101(00)00030-7 11312114

[B32] Fouque-Aubert A Chapurlat R Influence of RANKL inhibition on immune system in the treatment of bone diseases Joint Bone Spine 2008 75 5 10 10.1016/j.jbspin.2007.05.004 17920324

[B33] Simonet WS Lacey DL Dunstan CR Kelley MC Hang MS Luthy R Nguyen HQ Wooden S Bennett L Boone T Shimamoto G DeRose M Elliott R Colombero A Tan HL Trail G Sullivan J Davy E Bucay N RenshawGegg L Hughes TM Hill D Pattison W Campbell P Boyle WJ Osteoprotegerin: a novel secreted protein involved in the regulation of bone density Cell 1997 89 309 319 10.1016/S0092-8674(00)80209-3 9108485

[B34] Lacey DL Timms E Tan HL Kelley MJ Dunstan CR Burgess T Elliott R Colombero A Elliott G Scully S Hsu H Sullivan J Hawkins N Davy E Capparelli C Eli A Qian YX Kaufman S Sarosi I Shalhoub V Senaldi G Guo J Delaney J Boyle WJ Osteoprotegerin ligand is a cytokine that regulates osteoclast differentiation and activation Cell 1998 93 165 176 10.1016/S0092-8674(00)81569-X 9568710

[B35] Khosla S Minireview: the OPG/RANKL/RANK system Endocrinology 2001 142 5050 5055 10.1210/en.142.12.5050 11713196

[B36] Eagan T Ueland T Wagner PD Hardie JA Mollnes TE Dams JK Aukrust P Bakke PS Systemic inflammatory markers in COPD: results from the Bergen COPD Cohort Study Eur Respir J 2010 35 540 548 10.1183/09031936.00088209 19643942

